# The development of a brief parenting intervention to improve children's understanding of emotions: A Delphi study

**DOI:** 10.1111/papt.70043

**Published:** 2026-02-12

**Authors:** Sarah Lavender, Chris Hobson, Cerith Waters

**Affiliations:** ^1^ School of Psychology Cardiff University Cardiff UK

**Keywords:** child, Delphi study, intervention, mental health, parenting

## Abstract

**Objectives:**

Early emerging emotional and behavioural problems can have a significant adverse impact on children's wellbeing, mental health and educational outcomes that can persist through adolescence and adulthood. A growing body of research highlights children's ability to recognise and understand emotions as a transdiagnostic intervention target. Interventions aimed at helping parents support this ability in their child could lead to benefits. Therefore, the current study aimed to systematically develop a brief parenting intervention to support children's emotional understanding.

**Design:**

We followed Medical Research Council (MRC) guidance for developing complex interventions and used the Delphi method to gather consensus on the content and delivery of the intervention.

**Methods:**

In Round 1, interviews were conducted with academics, clinicians, parents and child and family practitioners with relevant experience. Thematic analysis of interviews generated themes and subthemes which were used to create a survey. In Round 2, this survey was shared with participants and they rated how important different potential elements of the intervention were to include in the manual.

**Results:**

Round 1 interviews generated themes including practicalities, creating a safe group space and intervention content. In Round 2, all participants (response rate: 86.4%) were asked to complete the survey and results indicated all items achieved at least moderate consensus for inclusion.

**Conclusions:**

How results were used to inform the intervention is discussed and implications for clinical practice addressed. This research has informed the development of a new parenting intervention which will be further researched in a feasibility trial.

## INTRODUCTION

Emotional and behavioural problems in childhood predict adverse educational, physical and mental health outcomes through late childhood, adolescence and into adulthood (Birmaher et al., 2004; Kim‐Cohen et al., 2003; Maughan & Collishaw, 2015; Roza et al., 2003). These difficulties can have a significant impact on children and young people's (CYP) wellbeing, health and education (Collishaw, 2015) and are a substantial and increasing cause of worldwide disease burden (Murray et al., 2012; Patton et al., 2016). Approximately one in eight CYP aged 5–19 years old met criteria for a mental diagnosis, an increase from one in ten in previous years (Sadler et al., 2018). Many children experiencing mental health difficulties do not receive a clinical diagnosis (Patton et al., 2016); therefore, these figures are likely to be an underestimate of need. Further, prevalence of mental health problems in CYP has risen since the Covid‐19 pandemic due to the potential long‐term impact of increased social isolation from peers and disrupted educational input (e.g., Adegboye et al., 2021; Cost et al., 2021; Mohler‐kuo et al., 2021; Nonweiler et al., 2020; Tong et al., 2025).

Due to the increasing prevalence of mental health problems in CYP, and research indicating that disadvantaged families are disproportionately affected by spending cuts to health and social care provision (e.g., reduced health coverage, restricted access to care), there have been resultant calls for preventative programmes to address early emerging behavioural and emotional difficulties as a public health priority (Collishaw et al., 2019; National Assembly for Wales, Children, Young People and Education Committee, 2018; Collishaw & Sellers, 2020). However, given the financial constraints on services, there is a need for cost effective and easily accessible preventative interventions to address emerging emotional and behavioural difficulties in CYP, before problems become more entrenched.

Behavioural and emotional problems in childhood are associated with difficulties in recognising and understanding emotions (Morris et al., 2010; Olson et al., 2011). Mier et al. (2010) suggest emotion recognition is the prerequisite that allows the recognition of intentions, which leads to improved interpersonal interactions. Krueger and Eaton (2015) suggest deficits in parent and child emotional competence are a transdiagnostic contributor to mental health difficulties. There is evidence suggesting that accurate emotion recognition facilitates positive social interactions, with difficulties in emotion recognition predicting elevated behavioural problems (Izard et al., 2001; Wells et al., 2020). Directly teaching children to learn about emotions can have beneficial effects on interpersonal skills (empathy and interpersonal problem solving, e.g., Webster‐Stratton & Reid, 2003) and behaviour problems (Wells et al., 2020).

A child's ability to recognise emotions is thought to develop in the context of the child–caregiver relationship (Asen & Fonagy, 2012). When such relationships are marked by attachment security with a caregiver who encourages emotional expression, the child is more likely to learn to better recognise and understand their own and others' emotions (Slade et al., 2005). Eisenberg et al. (1998) also highlighted that a parent's expression of emotions and their reactions to their child's emotions directly impact on children's understanding of emotions. There is a large body of literature to support this, and it has therefore been suggested that parental emotional understanding may be a critical target for intervention in childhood emotional and behavioural difficulties (Hajal & Paley, 2020).

Parental reflective functioning (PRF) involves the ability of a parent to be aware of their own emotions and behaviour alongside understanding their child's mental states and behaviours (Fonagy et al., 1991). Camoirano (2017) reported in a narrative review of the literature that PRF was associated with the quality of caregiving and children's level of attachment security, promoting children's capacity for emotional regulation. Parents with higher PRF have been shown to be more able to experience difficult and emotionally activating relational exchanges without becoming overwhelmed (Borelli et al., 2016). Mentalisation‐informed interventions have been developed to target PRF and are gaining increased research support (e.g., Byrne et al., 2020; Midgley & Vrouva, 2013). These interventions have been found to improve PRF in foster/adoptive parents, mothers with mental health and/or substance misuse difficulties, and parents of children with a diagnosis of Autism Spectrum Disorder (Adkins et al., 2018; Byrne et al., 2019; Enav et al., 2019; Midgley, Besser, et al., 2019; Midgley, Cirasola, et al., 2019; Suchman et al., 2008, 2016). These interventions have been delivered in individual, group or with blended individual and group formats and have shown positive impacts on PRF and parent and child outcomes. Lavender et al. (2023) in a systematic review of the literature for group delivered mentalisation‐based parenting interventions found many interventions led to improvements in PRF, the findings for child outcomes were mixed, with some studies showing improvements in child emotional and behavioural problems and others not.

Other parenting interventions promoting emotional learning have proved to be successful (Havighurst et al., 2020). Tuning into Kids is a 6‐week (2 h sessions) emotion‐focused group parenting programme developed in Australia, with a developing evidence‐base in terms of positive effects on parents' emotional awareness and emotion coaching skills, decreased emotionally dismissive parenting, increased child emotion knowledge and reductions in parent and teacher reported child behaviour problems (Havighurst et al., 2009, 2010, 2013, 2015). Similarly, other parenting interventions focused on emotion learning or regulation have been shown to be effective in reducing parental emotional distress, negative parenting practices and child behaviour problems, such as inattention, hyperactivity and emotional lability (Gaviţa et al., 2012; Herbert et al., 2013; Mason et al., 2016).

These interventions targeting parent and/or child emotion recognition, understanding and regulation tend to be quite lengthy, targeted at clinical or specialist populations, and require a high degree of facilitator training. Given the need to make the most effective use of available resources (Aylward et al., 2013), there is a need for an evidence‐based, easily accessible and relatively brief parenting intervention to help parents support their children to learn about emotions. An intervention such as this could be defined as a complex intervention: an intervention with interacting components that impact upon how the intervention will lead to a desired outcome (Craig et al., 2013). Complex interventions present difficulties both practically and methodologically. The Medical Research Council (MRC) framework (Craig et al., 2013) for developing and evaluating complex interventions highlights the importance of the development phase of new interventions that aims to enhance intervention effectiveness. The MRC framework also stipulates that stakeholders should be involved during all stages of the intervention development and evaluation process. The Delphi Method is an approach that can be used to gather opinion and consensus (Keeney et al., 2011) and has been used in the development of complex interventions (Domoney et al., 2020).

The current study aims to develop a brief and accessible parent‐focused intervention that aims to help parents support their child's emotional understanding. It will focus on the systematic development of an effective intervention by incorporating the views of key groups including academics and clinicians with expertise in child and adolescent mental health, parents of children identified as experiencing early emerging behavioural and emotional problems, and child and family facilitators who routinely work with children in early‐to‐middle childhood and their parents. In line with recommended guidance, the study has included a broad range of participants during the development phase of the intervention (Craig et al., 2013; Surowiecki, 2004).

## METHOD

### Design

In this study, a two‐round Delphi method was used. The Delphi method is used to generate qualitative data, is exploratory in nature and involves gathering opinion and generating consensus. The assumption is that the value of multiple opinions is greater than individual opinion (Habibi et al., 2014). The Conducting and Reporting Delphi Studies (CREDES) guidance (Jünger et al., 2017) informed the methodology, analysis and reporting of outcomes. A UK University Ethics Committee granted ethical approval for the study (EC.19.11.12.5756R2).

In line with widely accepted guidance, a mixed methods Delphi approach was used (Hasson et al., 2000). Qualitative data was collected via semi‐structured interviews. The interviews from the three groups of participants were then analysed using inductive Thematic Analysis to generate themes and subthemes for each of the three groups of participants. The subthemes from all three groups were then combined and transformed into summary statements that formed a survey that was sent to all participants in Round 2. Statements were rated in terms of importance to include or consider in the intervention on a 7‐point Likert scale. A similar methodology has been utilised in previous intervention development research (Domoney et al., 2020). All participants gave full informed consent to take part in the research.

### Participants

#### Clinicians and academics with expertise in child and adolescent mental health

Clinicians and academics with considerable experience in working with parents and children were recruited through a snowball sampling technique. This included identifying authors in the literature and utilising the professional networks of the research team. Twenty clinicians and academics were contacted, and eleven (all females of white ethnicity) agreed to participate (55%). The participants included an Educational Psychologist, three academics with expertise in parent–child relationships and/or emotional development, five Clinical Psychologists working across different regions of NHS Wales, a non‐UK Clinical Psychologist who has developed an emotion‐focused parenting intervention and a Psychotherapist trained in Mentalisation‐Based Treatment for Families (MBT‐F).

#### Parents

Parents were recruited through the Neurodevelopmental Assessment Unit (NDAU) at Cardiff University (a research study of children aged 4–8 with emotional or behavioural difficulties). Eight parents were contacted, and six (five female and one male, all White British ethnicity) consented to take part and were interviewed (75%).

#### Child and family intervention facilitators

Child and Family Intervention Facilitators were health and social care staff with experience in delivering parenting groups without formal professional psychology or social work registration. They were recruited through a snowball sampling technique. Three Family Support Workers, two Graduate Mental health workers and two Assistant Psychologists were interviewed. All participants were females of white ethnicity.

### Procedure

The MRC guidance (Craig et al., 2013) on developing complex interventions was used as a framework for developing the intervention. The first stage involved examining existing evidence. A literature review using the terms Parent* intervention emotion*, Parent* training emotion*, Parent* group emotion*, Parent* intervention mentalis* and Parent* training mentalis* was conducted. The review identified 21 papers that described 13 different parenting interventions. All interventions tended to be lengthy (at least 6 weeks long), required a high degree of facilitator training and were targeted at specific populations such as foster carers, adoptive parents and depressed mothers. Please see Data S1 for a summary table of these studies and interventions. This highlighted the need for a brief intervention focused on helping parents to support their children to understand emotions.

The second stage of intervention development entailed the production of a portfolio of information, designed by two clinical psychologists with relevant experience of mentalisation or emotion‐focused interventions with children and families. The portfolio described the theoretical rationale, broad aims, basic proposed set‐up and a brief proposed outline of the programme content. The portfolio was intended as a “starting point” to enable and structure discussion in the Round 1 interviews. This portfolio was firstly sent to the Clinicians and Academics group, and semi‐structured interviews were then conducted via video conferencing platform Zoom to canvass their views.

The third stage of developing the intervention involved semi‐structured interviews with Parents and Child and Family Intervention Facilitators. These participants were provided with the same intervention portfolio and were subsequently asked questions in a semi‐structured interview format via the video conferencing platform Zoom. See Appendix A for interview schedules for the semi‐structured interviews with the three groups.

In the final stage, interviews from each of the three groups were analysed, subthemes were combined and transformed into summary statements which were then used to create a survey, using the Qualtrics Survey Software. The survey asked participants to rate the importance of each item for inclusion in the intervention. The time between beginning the Round 1 interviews and disseminating the Round 2 survey was 6 months.

Following these stages, the intervention materials were developed: a manual for facilitators and corresponding workbook for parents.

### Data analysis

Thematic Analysis (Braun & Clarke, 2006) was used to analyse the data. Separate analyses for the three groups of participants were conducted to ensure themes across groups were not conflated. An inductive approach was used, and the data were coded in relation to semantic content.

Interviews were transcribed and read twice to generate initial codes by the first author. Codes were assigned and sorted into potential themes. Following discussion between authors, themes were finalised and summarised and quotes to exemplify the themes were selected. The process was conducted using the NVivo 12 qualitative data analysis software package.

For the second round, subthemes from the three analyses were transformed into summary statements that participants rated on a Likert Scale of 1–7 ranging from “unhelpful to include/consider” to “essential to include/consider”. Consensus on a summary statement was achieved if 60% of respondents marked within two adjacent response points on the Likert Scale. This method and level of consensus have been used in previous Delphi studies that aimed to develop a mental health intervention (Domoney et al., 2020; Pezaro & Clyne, 2015). There is no single definition of consensus across Delphi studies (Jorm, 2015). Therefore, to understand different levels of consensus it was agreed that statements that achieved between 60% and 79.5% consensus would be classified as moderate consensus and statements achieving between 79.6% and 100% would be classified as high consensus.

Items that reached consensus were included in the intervention materials. Similarly, if consensus was reached for items to not be included, they were not considered. For statements where 60% consensus was not achieved, the researcher planned to return to the interview transcripts and previous knowledge of the literature to consider whether the item was important to include or consider in the intervention.

### Quality assurance and reflexivity

Triangulation and Respondent Validation are two methods of assessing validity in qualitative research (Mays & Pope, 2000). The method of triangulation was used in the current study as participants from different groups (sources) holding different perspectives were interviewed. This allowed the researcher to look for patterns of convergence and divergence across groups. In addition, the Round 2 Survey acted as a form of respondent validation as it allowed participants to provide anonymous feedback on the themes and subthemes generated during Round 1 as well as combining data gathered from the three different groups.

As a White Female Trainee Clinical Psychologist from Britain, the author held westernised assumptions about emotions and emotional expression. In addition, the author had experience of Mentalisation Based Theory and treatment and Attachment based therapies. These factors may have influenced the initial information given to participants, the author's understanding of what was spoken about during interviews and the analysis process. The author attempted to remain open in their questioning and responses to interviewees. In addition, experts working in a diverse range of settings using different psychological theories and models were recruited. There was a risk that participants may simply acquiesce to the information presented in the intervention outline; therefore, the author took an active approach to encourage criticism and ensure that they were not defensive of the initial intervention outline.

## RESULTS

### Round 1: Interviews

**FIGURE 1:**
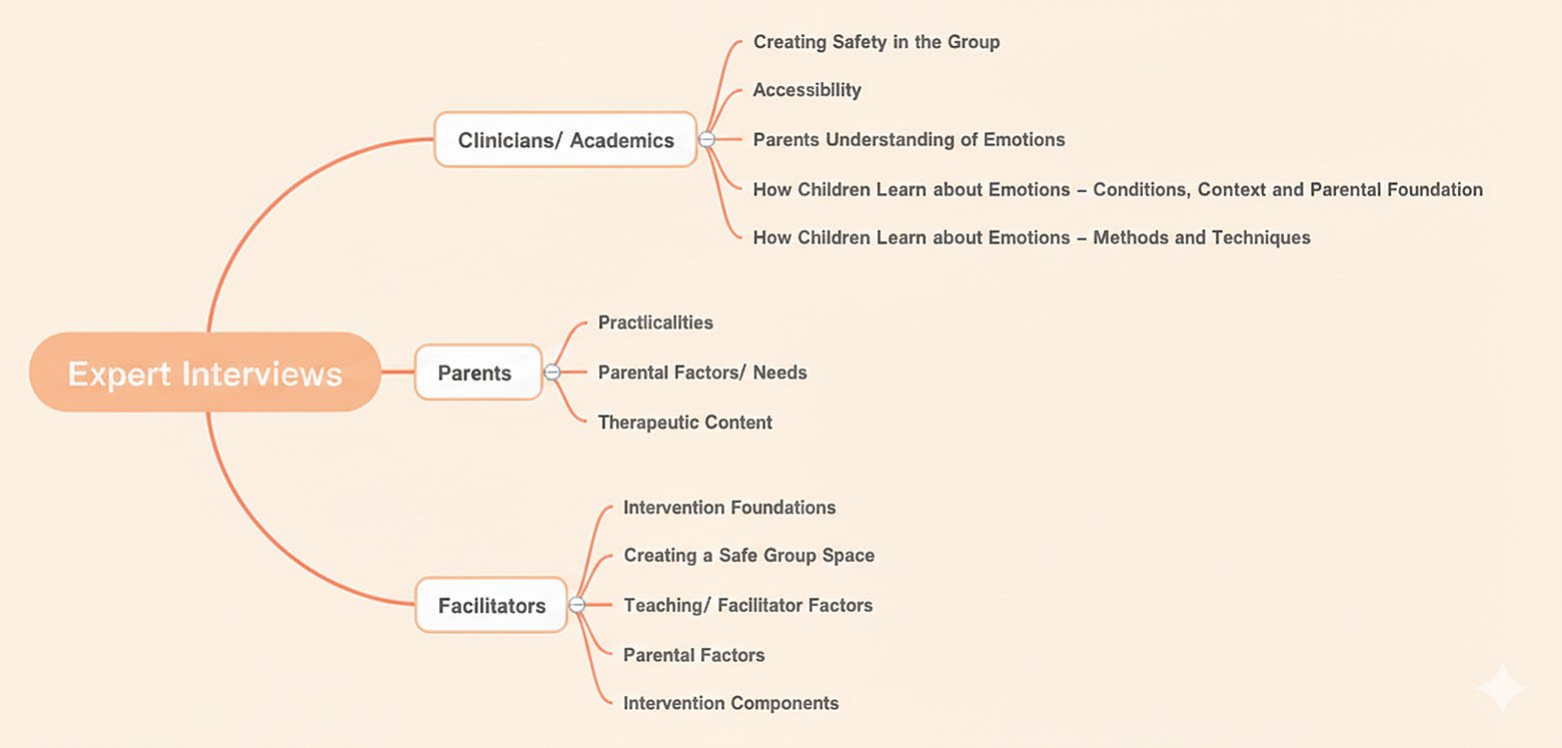
Themes from interviews with clinicians/academics, parents and facilitators.

Figure 1 shows the themes identified in the analyses of the three participant groups: Clinicians and Academics, Parents and Facilitators.

#### Clinicians and academics with expertise in child and adolescent mental health

Table 1 outlines themes and subthemes with relevant quotes identified in the analysis of the interviews with clinicians and academics with expertise in child and adolescent mental health. The first theme of creating safety in the group environment was identified by all clinicians and academics. They identified a number of components that facilitate safety including support from those with similar experiences, a non‐blaming approach and skilled facilitators. In addition, parents having access to additional support if needed was also highlighted as important.

**TABLE 1:** Themes and subthemes with relevant quotes from interviews with clinicians and academics with expertise in child and adolescent mental health.

Themes/subthemes	Quotes
Creating safety in the group	
Relationship and support from other parents	“So that's another aspect of group‐based programs, they obviously have that support from other parents, they can see that they're not the only person going through these sorts of challenges so that aspect of having social support which they might not have otherwise” (Clinician/Academic 10)
Non‐blaming/judgmental of parents	“A learning environment where being able to make a mistake is ok, um that sense of I feel really strongly whatever intervention we're doing but particularly in talking to parents because it's so easy to shame and blame parents” (Clinician/Academic 3)
Trust in facilitator	“Delicate balance of trusting the person enough to think that they've got enough expertise to be, to know their stuff about this and trust them but not wanting it to feel too much of a gap” (Clinician/Academic 1)
Ongoing engagement and holding in mind	“You may need a facilitator around if anything comes up afterwards or during the week, is there any way of contacting a facilitator if some difficult things come up for you” (Clinician/Academic 3)
Accessibility	
Accessible language and content	“Would very young parents be able to understand this and would it be accessible um so thinking about the reading age in different areas. I think you know there's already a lot of what we do in the Psychology team is, I feel inaccessible for lots of the families that I work with” (Clinician/Academic 2)
Accessible timings and location	“One of the things that we find quite difficult with group based programs is finding a good time for parents” (Clinician/Academic 1)
Increased accessibility of skills through active participation	“So I think a lot of experiential work in the actual sessions with the parents themselves sort of role playing or talking through scenarios or watching scenarios and thinking about what that brings up for them” (Clinician/Academic 4)
Practical strategies and resources for parents	“Practical techniques so they feel they can go away with something tangible”(Clinician/Academic 11)
Engagement difficulties working online	“We've done things with parents they're often on their iPhone which cuts the screen or the connections not very good or there's stuff going off in the background, which then distracts them or children are coming in so you know it's not perfect” (Clinician/Academic 9)
Engagement advantages working online	“I think the online space is working really well with our (name of intervention) programme so I think that's really viable” (Clinician/Academic 8)
Cultural considerations	“You have to think about cultural context there, um, and that is a bit of a Eurocentric white western way of ascribing and thinking about attachment and emotions, relationships” (Clinician/Academic 10)
Parents understanding of emotions	
Intergenerational patterns	“Their own experiences of being parented and own history of being parented is really important being able to look at that and then seeing I guess how that gets played out in the here and now with their own children” (Clinician/Academic 4)
Parental emotional literacy	“I bet there are parents who are themselves, are confusing certain emotions, particularly the negative emotions so I think that it is, it's a very, emotion recognition it's very basic it's very simple skill but it just has to be taught” (Clinician/Academic 5)
Parental ability to understand and regulate their own emotions	“You actively work with the parent on thinking about their own emotional states, so when you come to thinking about a child's behavioural problem, or a difficult situation, you're not focusing on what the child is thinking or feeling when they're having a temper tantrum in the supermarket, you're thinking about what the parent is feeling, and you're helping them to make sense of their own emotional responses” (Clinician/Academic 6)
Learn about emotions – Conditions, context and parental foundations	
Parental acceptance of children's emotions	“The parents have to be able to respond in a way that communicates that it is ok to feel these feelings and that it's ok to talk about feelings” (Clinician/Academic 8)
The earlier developmentally the better	“It could be delivered to younger children because they'll be going through that process of getting to understand emotions anyway” (Clinician/Academic 10)
Importance of safe and warm parental relationships to facilitate emotional learning	“The more the parent is attentive and responsive to the child's needs the more they're going to feel emotionally regulated and by feeling emotionally regulated they're probably then more likely to identify different emotions and to express them as well and to have a language around emotions” (Clinician/Academic 11)
Parental reflective functioning	“What I think is key in parenting programs is developing reflective functioning for parents, so having a sense of look I can accurately guess your psychological state and reflect that back to you and give you an impression that will help you to know that we have different minds, separate minds, separate psychological states” (Clinician/Academic 6)
Context – system/societal understandings	“For me it's about also recognising that parenting takes place in a wider context, that my capacity to engage with my child or the manner in which I engage with my child is going to be heavily influenced by work place pressures, my quality of relationship with my partner, whether or not I have other aspects of social support that are impinging to either promote or undermine my parenting” (Clinician/Academic 7)
How children learn about emotions – Methods and techniques	
Embedding emotional talk in everyday life	“Teach them and work with them through play, is often the best way to do it so, um, using toys, using natural situations that occur, rather than trying to sit down and teach it” (Clinician/Academic 9)
Making space for curiosity and reflection	“Having an adult too, um, name it, help you understand it, make their best guess but not impose their feeling or, um, on the child. So like lots of ‘I wonder if this has happened’ and that's why you're feeling like that?’” (Clinician/Academic 2)
Emotional attunement	“Noticing when children are coming to you for comfort, and when they need help to organise their feelings, and when they need support to learn to play to do things for themselves, and have that support to be independent, and that's key to emotional development” (Clinician/Academic 2)
Naming emotions and modelling	“They might not necessarily have the language or the labels to put on it but just to start priming them about emotions at that early age will just help, it becomes a common universal language that they can relate to with all adults” (Clinician/Academic 11)
Deepening parental understanding of behaviour	“Making the link that an emotion, is not just a feeling of arousal internally, but it has communication so that is how we begin, and how this is important for development, and how it is linked to relationships and empathy” (Clinician/Academic 5)

The second theme identified by all clinicians and academics highlighted the importance of ensuring all aspects of the group were accessible for all parents. This included accessible content as well as practicalities such as timings and location. They also highlighted active participation as important for increasing accessibility and providing practical strategies and resources. Advantages and disadvantages of working online were also highlighted. Cultural considerations were also raised as important in ensuring that the group was accessible to all parents.

The third theme identified was about parents increasing their understanding of emotions as an important element of the intervention. Many highlighted the importance of raising intergenerational patterns as a factor that influences parents' understanding of emotion. However, there was some concern about parents feeling safe enough to do so in a short intervention. Parental emotional literacy and ability to understand and regulate their own emotions were identified as essential in allowing parents to teach their children about emotions.

Learning about emotions – Conditions, context and parental foundations was the fourth theme identified. Clinicians and academics were able to provide a wealth of information about how children best learn about emotions. Parental acceptance of children's emotions, both positive and negative, was highlighted. It was also suggested that providing parents with the skills as early as possible to help their child learn about emotions, then the better for the child's development. The importance of safe and warm parental relationships and PRF were also identified as important to allow parents to help their child learn about and understand emotions. Systemic and societal narratives around emotion were raised as contextual factors influencing how successful an intervention may be.

The final theme identified was more specifically about methods and techniques that facilitate a child's emotional learning. Embedding emotional talk in everyday life and making space for curiosity and reflection were raised as important for helping children to recognise and understand their own and others' emotions. The importance of emotional attunement, naming emotions and modelling appropriate responses to emotions was also highlighted. Many participants spoke about the need for deepening parental understanding of their own emotions and behaviour to better understand their child's behaviour. Participants also identified the importance of helping parents identify the emotion underlying their child's behaviour and then supporting their child to understand the links between their emotions and behaviour.

#### Parents

Table 2 outlines themes and subthemes with relevant quotes identified in the analysis of the interviews with parents. The first theme identified by all parents was practicalities including timings, location and size of the group. The benefits and drawbacks of online delivery were also highlighted. Parental factors/needs was the second theme identified as important including support from other parents, reduction of self‐blame and considering how they were parented as well as wider global impacts on parenting. The final theme was about therapeutic content where parents highlighted the importance of communication, understanding what is going on underneath children's behaviour and developing practical strategies to aid them in helping their child to understand their own and others' emotions.

**TABLE 2:** Themes and subthemes with relevant quotes from interviews with parents.

Themes/subthemes	Quotes
Practicalities	
Parents juggling time	“I work full time and I work long hours” (Parent 1)
Comfortable sized group	“Yeah not too big and not too small so it feels really intense, um and too big you get lost so sort of a balance” (Parent 2)
Accessible and relaxed setting	“I think if it felt a bit more if it was a bit more of an informal environment, I think it would be easier to speak rather than you know if it was right attend the university classroom so to speak it would be more, I think it would be better in a more relaxed environment” (Parent 4)
Online accessibility	“It saves you getting a babysitter you're usually at work all day so you can do it in the evening and obviously geographically as well you can have people from all areas instead of just looking at a small area for people to travel to um so yeah it works really well in terms of accessibility” (Parent 2)
Online reduces human connection	“I think it's harder to connect with someone online, unless it's sort of like a one on one or like just a few little people. I think if you've got a group of maybe 10 parents you wouldn't get to know them on that personal level, you know sort of chit chat that you would normally have in between like a tea break” (Parent 1)
Parental factors/Needs	
Importance of parental peer support	“to meet peers for myself as well as for the children so yeah they're really good they're invaluable really” (Parent 3)
Parental self‐blaming	“be aware of maybe shame and guilt from the parent's point of view” (Parent 2)
Awareness of how parents were parented	“the way you were parented does hugely impact the way you parent your children, so yeah, I think that needs to be done in a sensitive way” (Parent 3)
Understanding the impact of global change and events on parenting	“I think it's just because of the world we live in they're emersed in the world of technology you know they're spoken to like, well they are they're little people but their spoken to like adults almost you know, they're expected to understand so much more and I think it's giving them it's almost giving them that sort of understanding of it with that comes a lot more responsibility on their emotions” (Parent 1)
Therapeutic content	
Encouraging communication	“I think that's the biggest thing with (child's name) is encouraging communication more than ever not to bottle things up” (Parent 4)
Developing emotional talk	“our children often really don't know can't name their emotions although they are feeling the emotion” (Parent 2)
Understanding what's underneath child's behaviour	“he wanted a tiny little sticker and you just think it's a tiny little sticker but he really wanted it and it was hard to calm him down but I think it was so much more than just this tiny little sticker” (Parent 3)
Developing practical strategies	“Tool kit of strategies to use to help um de‐escalate some behaviours” (Parent 4)
Manageable home tasks	“Making them accessible flexible short and um sort of scaffolding what you want the parents to do” (Parent 2)
Finding what works ‐ recognising difference and changes over time	“I suppose just not every child's the same either so like it might work brilliantly for one child but for another it just doesn't… we've used so many different methods over the years, and some of them worked, and then they stopped working” (Parent 1)

#### Child and family intervention facilitators

Table 3 outlines themes and subthemes with relevant quotes identified in the analysis of the interviews with child and family intervention facilitators. The first theme identified by facilitators was intervention foundations that are important prior to delivering a parenting intervention. Facilitators felt that the intervention should be universally offered to parents facing similar difficulties. They also highlighted that an accessible community location with good transport is very important. As with other participant groups, considerations specific to online versus in‐person delivered interventions were highlighted.

**TABLE 3:** Themes and subthemes with relevant quotes from interviews with child and family intervention facilitators.

Themes/subthemes	Quotes
Intervention foundations	
Universally offered – all parents can benefit	“People are all going to find a benefit from it because we've all got, every child has got emotions” (Facilitator 4)
Maximising connection – parents facing similar difficulties	“It could be so varied that actually the whole point of a group where connection is supposed to happen rapport and seeing similarities that wouldn't happen as easily…. I would say that more targeted would bring a better connection between people” (Facilitator 6)
Transport – accessible location	“Definitely transport and a venue would be the biggest hurdles I think” (Facilitator 1)
Online more accessible and comfortable	“Those parents or carers not having to drive, not having to get anywhere they're just already at home, it removes that anxiety of having to leave your house you know for a lot of people that's huge isn't it having to go to a group and it feels safer I think for people in their home” (Facilitator 4)
Face‐to‐face more connected and nurturing	“I think obviously my personal preference would be to do it face‐to‐face because you get all the nurturing and everything that comes from it” (Facilitator 1)
Community (not clinical) relaxed setting	“Community based, so not coming into a hospital, not coming into like a clinic setting, um, I think makes it feel very health focused, and I think that's kind of scary and off‐putting” (Facilitator 6)
Parental factors	
Parent understanding that they can facilitate change	“When we're called in they would like us to change their child rather than realising that they've got the power to change things themselves” (Facilitator 1)
Complexities of confidentiality	“People knowing each other and going through confidentiality that has come up a few times um which can be really difficult because sometimes it's just kind of a oh I'm not going to that group point blank because I know this person” (Facilitator 7)
Parental availability	“People have got so much going on in their everyday lives” (Facilitator 6)
Preparing parents for difficult content	“I would actually warn them and say that session the week before next week we're going to be talking about this, this is what it's going to entail so you prepare them” (Facilitator 3)
Teaching/Facilitator factors	
Knowledge of psychological development	“I think it's more about the skills that someone can bring, so if they're able to sort of know the background and the theories, I guess behind what we're teaching” (Facilitator 6)
Supportive team around them	“Really good team you work with so you are able to have clear goals and to make sure you always feedback you know and reflect on things” (Facilitator 5)
Ability to show fallibility/normalise	“I'm really open as well, and say ‘I do lose it sometimes’, you know, none of us are perfect” (Facilitator 1)
Skilfully manage group dynamics	“I suppose it's about managing it and facilitating your group really when you get chatty big strong characters you can um keep them at bay if that makes sense without being disrespectful” (Facilitator 2)
Use interactive and engaging approaches	“Well so kind of that interactive kind of what are they called like menti meters and you know things that they can input so that it feels um not as kind of talked at I suppose because I think sometimes that can be difficult” (Facilitator 7)
Combine learning, informality and fun	“Learn through fun having fun and learning without knowing they're learning” (Facilitator 5)
Connecting with parents	“I guess it's really connecting with the parents in their struggles you know what I mean showing that we get it” (Facilitator 4)
Creating a safe group space	
Containment and empathy	“They're able to say oh my god I've had a really pants week this week and he's done that and he's done that. They're able to have that containment and they go home and are then able to then parent a bit more effectively” (Facilitator 2)
Connecting with parental peers	“I'm just thinking about our parents and a lot of them seek just the fact of meeting another parent going through similar difficulties um can be so much more reassuring and empowering” (Facilitator 6)
Creating a supportive network outside of sessions	“Checking in with you even if it's just a text just for reassurance to say oh you know how is it going, sometimes they take kindness to that you know rather than just rocking up the week after in the next session” (Facilitator 3)
Facilitating parental solutions	“I think it's a massive, um, message when it comes from somebody who has actually done it. I think that's the way forward” (Facilitator 3)
Intervention components	
Accepting/normalising emotion	“It's alright to feel these things… you're not allowed to feel jealous, you're not allowed to feel angry, you're not allowed to feel those negative feelings but actually it's alright to feel those and express them and deal with them, there's no point in keeping them inside” (Facilitator 2)
Developing empathy for the child	“Help them understand a little bit that children have a world of their own and their own language it's really important for us all to leave ours and live in theirs and come back” (Facilitator 5)
Linking emotions to bodily sensations	“Get them to understand what's going on in our heads but also in our bodies” (Facilitator 7)
Giving parents emotional vocabulary	“Developing emotional literacy and emotional vocabulary so for the parents to see the behaviour that they may or may not like but to express that vocalise it, so that the parents have got words or ways to communicate their behaviour rather than show it, so the emotional vocabulary would be the starting point” (Facilitator 1)
Simple/accessible at‐home tasks with emphasis on reflection	“We're very informal about it, so we're like ‘just see how it goes’, you'll be feeding back in, but we don't want to be too formal, so you know they don't come back to group” (Facilitator 2)
Understanding behaviours more deeply	“Especially when behaviour comes in with the children, they see the management of behaviour and the understanding of behaviour as still two slightly different things” (Facilitator 1)
Parents understanding own emotions and regulation	“So for instance asking them to explore their feelings what riles them up or what upsets them and how they deal with it helps them to relate it to their children. It's a bit of a light bulb moment sometimes” (Facilitator 2)
Practical resources	“We very much gave them the equipment to use at home so they went home basically with a goody bag of stuff um so that wasn't a barrier” (Facilitator 4)
Parents learning to help child regulate emotions	“Then being able to go, and you know, do some exercise, or go and draw, or go and play, or whatever it is to regulate it, and I sort of continuously say that it's about that role modeling” (Facilitator 7)
Highlighting transgenerational effects	“It's made them reflect and they're already thinking about how their parenting effected them” (Facilitator 3)

Facilitators identified a second theme of parental factors important to consider in the intervention which included parents understanding that they can facilitate change for their child. The complexities around confidentiality and parental availability were also highlighted. Preparing parents for difficult content was also raised as an important consideration in order for parents to feel safe and supported.

A third theme identified was teaching/facilitator factors including the skills needed to effectively deliver parenting interventions. Knowledge of psychological development, a supportive team, the ability to show fallibility/normalise distress and being able to skilfully manage group dynamics were all identified as important. Participants described the need to use interactive and engaging approaches during intervention delivery, combining learning, informality and fun to increase engagement and safety. Facilitators also mentioned the importance of connecting with parents to enable safety, openness and full participation in a group‐delivered intervention.

Linked to the subtheme of connection, facilitators highlighted a fourth theme of the importance of creating a safe group space for parents and the importance of this for engagement. Containment and empathy and connecting with parental peers were highlighted as important. Some participants spoke about this being a normalising and validating experience, as well as an opportunity for parents to learn from each other. Creating a supportive network outside of sessions was acknowledged as beneficial and the importance of facilitating parental solutions for difficulties faced by group members was considered valuable.

The final theme identified by facilitators was important intervention components. Including how accepting and normalising emotions and developing empathy for children's emotional experience are important skills for facilitators to model to parents. Participants also communicated the importance of linking emotions to bodily sensations and giving parents emotional vocabulary. Practical resources and simple at‐home tasks were also described. Developing parents' reflective skills was also indicated, including understanding behaviours and emotions more deeply. Examples included helping parents to better understand their own emotion regulation strategies and transgenerational effects within families.

### Round 2: Consensus survey

From the subthemes identified by the three groups in Round 1, summary statements were created. The summary statements from all three groups were combined and sent to all participants who were interviewed in Round 1, with 19 out of 22 (86.4%) completing the consensus survey in Round 2. Of the three participants who did not complete the Round 2 survey, one was a clinician/academic, one was a parent and the other was a family intervention facilitator. The summary statements and results are presented in Table 4. All items achieved the 60% consensus to be included or considered in the intervention. There was variation in the strength of consensus across the items; 58 of the 70 items achieved a high consensus (82.86%) and 12 achieved a moderate consensus (17.14%). How results from the Round 2 consensus survey were then used to inform the development of the intervention materials is detailed in the discussion.

**TABLE 4:** Summary statements with means, standard deviations, mode maximum/minimum scores and % consensus achieved.

Themes	Summary statement	Mean	SD	Mode	Min	Max	% consensus
Creating safety in the group	Facilitating the development of supportive relationships between parents that attend the group	6.63	1.13	7	3	7	94.44
	2 Adopting a non‐judgmental and non‐blaming approach	7.50	1.03	7	3	7	94.44
	3 Parents developing trust in the facilitator	7.25	0.86	7	4	7	94.44
	4 Facilitators making contact with participants between intervention sessions to encourage engagement	5.31	1.18	5	2	7	77.78
Accessibility	5 Ensuring that language and content is accessible	7.44	0.70	7	5	7	100.00
	6 Ensuring timings and location are accessible	7.06	0.96	7	4	7	94.44
	7 Encouraging active participation in the intervention, e.g., through role play, experiential exercises, group discussion	6.44	1.36	7	2	7	88.89
	8 Providing practical strategies and resources for parents	7.19	0.85	7	5	7	100.00
	9 Acknowledging that engagement can be difficult for parents in an online intervention	6.75	0.97	5	5	7	100.00
	10 Acknowledging that an online intervention can be more accessible for some parents	6.50	1.06	5	4	7	88.89
	11 Ensuring consideration is given to parents heritage and cultural backgrounds	6.94	0.99	7	5	7	100.00
Parents understanding of emotions	12 Highlighting intergenerational patterns of parenting	6.13	1.25	5	3	7	83.33
	13 Developing parents emotional literacy, e.g., language around emotions	7.63	0.55	7	5	7	94.44
	14 Developing parents ability to understand and regulate their own emotions	7.38	0.86	7	4	7	94.44
How children learn about emotions – conditions, context and parental foundations	15 Providing parents with tools to be accepting of all children's emotions	7.13	0.84	7	4	7	94.44
	16 Delivering the intervention when the child is at an early developmental stage	5.75	1.08	5	3	7	77.78
	17 Highlighting and encouraging safe and warm parental child relationship to facilitate emotional learning	7.06	0.96	7	4	7	94.44
	18 Improving parental reflective functioning	7.13	0.84	7	5	7	100.00
	19 Acknowledging the impact of societal expectations on parents and children	6.50	1.00	5	4	7	94.44
How children learn about emotions – Methods and techniques	20 Providing parents with skills to embed emotional talk in everyday life	6.88	0.83	7	5	7	100.00
	21 Encouraging parents to make space for curiosity and reflection	6.69	1.11	7	4	7	88.89
	22 Encouraging emotional attunement between parent and child	7.13	0.97	7	5	7	100.00
	23 Encouraging parents to name emotions and model responses to emotions and coping with them	7.31	1.04	7	3	7	94.44
	24 Providing parents with skills to deepen their understanding of behaviour, e.g., emotions underlying behaviour	7.00	1.17	7	3	7	94.44
Practicalities	25 Appreciating the difficulties for parents juggling multiple demands on their time	6.38	1.28	7	3	7	83.33
	26 Ensuring that the size of the group is comfortable for all	6.44	1.07	5	4	7	88.89
	27 Providing an accessible and relaxed setting for the intervention	6.44	1.27	7	3	7	83.33
	28 Ensuring that the intervention can be delivered online as well as in person	6.44	1.07	5	4	7	88.89
	29 Acknowledging that there may be less human connection in an online intervention	6.56	1.04	7	4	7	88.89
Parental factors/needs	30 Ensuring parents have peer support from other parents in the group	5.81	1.20	5	3	7	72.22
	31 Highlighting and considering parental self‐blame	6.00	1.71	7	1	7	77.78
	32 Talking about how parents were parented themselves	5.94	1.67	7	2	7	66.67
	33 Highlighting the impact of global change and events on parenting, e.g., changes in technology, COVID‐19 pandemic and lockdown	5.81	1.54	5	2	7	72.22
Therapeutic content	34 Providing parents with skills to improve communication with children	6.81	0.94	7	5	7	100.00
	35 Developing parents ability to talk about emotions	7.00	1.11	7	3	7	94.44
	36 Helping parents to understand what's going on underneath their child's behaviour	7.06	0.89	7	5	7	100.00
	37 Providing parents with practical strategies to help children learn about emotions	7.06	0.83	7	5	7	100.00
	38 Setting manageable at‐home tasks	6.50	1.17	5	3	7	94.44
	39 Acknowledging individual differences and that the same thing will not work for everyone and may change with age	6.63	1.28	7	3	7	88.89
Intervention foundations	40 Offering the intervention universally so that all parents could benefit	5.69	1.39	5	3	7	61.11
	41 Offering the intervention to parents facing similar difficulties in order to facilitate connections	5.81	1.25	5	3	7	62.50
	42 Ensuring the intervention takes place in an accessible location with good transport links	6.13	1.10	5	3	7	88.89
	43 Acknowledging that engaging in an online intervention can be more accessible and comfortable, e.g., not needing to travel, having home comforts at hand etc.	5.81	0.99	5	3	7	83.33
	44 Acknowledging that a face‐to‐face intervention can be more nurturing and people can feel more connected	6.13	1.04	5	4	7	83.33
	45 Ensuring the intervention takes place in a community (not clinical) relaxed setting	6.19	1.10	5	4	7	83.33
Parental factors	46 Parents understanding that they can facilitate change in their child	7.13	0.84	7	5	7	100.00
	47 Addressing the complexities of confidentiality, e.g., group members knowing each other or sharing information outside the group	6.69	1.47	7	3	7	83.33
	48 Acknowledging that parents are juggling multiple demands	6.25	1.42	5	2	7	83.33
	49 Preparing parents for difficult content	6.13	1.15	5	3	7	83.33
Teaching/facilitator factors	50 Facilitators having knowledge of psychological development	6.44	1.32	7	3	7	83.33
	51 Facilitators having a supportive team around them	6.50	1.22	7	3	7	88.89
	52 Facilitators having the ability to show fallibility and normalise emotions and difficulties	6.69	1.21	7	3	7	88.89
	53 Facilitators being able to skilfully manage group dynamics	7.50	1.03	7	3	7	94.44
	54 Using interactive and engaging approaches	7.31	0.79	7	5	7	100.00
	55 Combining learning, informality and fun	6.44	1.18	7	4	7	83.33
	56 Facilitators connecting with parents	6.81	1.26	7	3	7	88.89
Creating a safe group space	57 Providing containment and empathy to parents	7.00	1.26	7	3	7	88.89
	58 Facilitate connection between parental peers	5.75	1.57	5	2	7	77.78
	59 Offering a supportive network outside of intervention sessions	5.25	1.41	5	2	7	66.67
	60 Facilitating parental solutions to difficulties, e.g., parents helping each other find answers to problems	6.06	1.54	7	3	7	72.22
Intervention components	61 Helping parents to be accepting of emotions and normalise children's experiences of emotion	7.25	0.86	7	5	7	100.00
	62 Developing parental empathy for the child	7.31	0.79	7	5	7	100.00
	63 Linking emotions to bodily sensations	6.75	0.97	5	5	7	100.00
	64 Giving parents emotional vocabulary	7.25	0.98	7	4	7	94.44
	65 Giving parents simple and accessible at‐home tasks with an emphasis on reflection	6.50	1.00	5	4	7	94.44
	66 Deepening parental understanding of their child's behaviour	7.19	0.98	7	4	7	94.44
	67 Helping parents to understand their own emotions and how to regulate these	7.06	1.02	7	4	7	94.44
	68 Providing practical resources for parents to take away and use	6.50	1.06	7	4	7	88.89
	69 Providing parents with skills to help their child regulate their emotions	7.06	1.02	7	4	7	94.44
	70 Highlighting transgenerational effects on parenting	5.81	1.38	5	3	7	66.67

## DISCUSSION

This study aimed to develop a brief and accessible parenting intervention to help parents support their child's emotional learning. Following development of an initial portfolio of information describing a proposed intervention, the first Round of this Delphi study involved interviews with clinicians and academics, parents and child and family intervention facilitators. Themes were generated regarding important aspects of the intervention. There was overlap in the themes generated by the different participant groups. All subthemes were transformed into summary statements, and all those who had initially participated in interviews were invited to rate all of the summary statements on the level of importance for inclusion in the intervention. Consensus on each of the items was then calculated.

### Practicalities

Across all three groups, themes related to the practicalities of the intervention were identified including accessibility of the content, manageable at‐home tasks and providing parents with practical strategies and resources. There was also high consensus in the survey for these themes across the three participant groups. Active and interactive methods of engaging parents were themes identified by clinicians, academics and facilitators that also achieved high consensus. Ensuring that the intervention is accessible in terms of timings and locations was also highlighted across groups, and again there was consensus for a community rather than clinic‐based setting. Facilitators and parents also highlighted the demands on parents' time and how this can pose a challenge for engagement. Therefore, the findings from the analysis suggested that these practical factors should be given considerable consideration in the development of the intervention manual. These findings are in line with previous research on successful parenting interventions (Adkins et al., 2018; Havighurst et al., 2009; Herbert et al., 2013; Webster‐Stratton, 2001).

Across participants, there were common themes around the benefits and challenges of delivering the intervention online versus in person. The accessibility of online interventions was acknowledged by all participant groups. Interviews were conducted post the COVID‐19 pandemic, and many participants discussed how their views about the benefits of digitally delivered parenting interventions had increased since the pandemic. However, there was a common theme that an online parenting group would lead to a reduction in the connection between parents and facilitators, which could decrease engagement. There was high consensus in the survey around the advantages and disadvantages of online working. Therefore, the analysis suggested that the intervention should be designed to be delivered *online or in person*, depending on service context and parental needs and preferences. There is recent research suggesting that online parenting programmes for children with emotional and behavioural problems can be effective (Florean et al., 2020). However, further research is needed to identify how this compares to in‐person interventions.

Finally, in relation to themes around practicalities, although the intervention was initially conceived as being targeted at children with emerging emotional and behavioural difficulties, the analysis showed that there was only moderate consensus about whether the intervention should be targeted in this way or offered universally. Hence, the analysis was not clear enough to suggest that the intervention should be offered universally, but did indicate that the level and type of difficulties did not need to be the same across all parents attending the intervention. It may be that the intervention could be of benefit to many parents and children but given limited healthcare resources it appears prudent that those experiencing difficulties receive support (Collishaw & Sellers, 2020; Department of Health and Social Care, 2017; National Assembly for Wales, Children, Young People and Education Committee, 2018).

### Creating safety

Creating safety in the group context was highlighted across all three groups of participants. Themes of being non‐blaming and non‐judgemental of parents, as well as showing empathy and providing containment, all achieved high consensus. Therefore, the analysis indicated that these components should be incorporated into the intervention manual and materials, whilst acknowledging that facilitator style will have an impact on the delivery of these therapeutic skills. The importance of this is less well documented in the parenting intervention literature. However, there is evidence that a stronger therapeutic alliance in group‐delivered parenting interventions leads to better outcomes (Schmidt et al., 2014).

Facilitator factors such as having knowledge of psychological development, an ability to show fallibility and skills in managing group dynamics all achieved high consensus. Hence, these intervention components should inform the recommendations for the facilitators' approach to the delivery of the intervention and will be included in the intervention manual and any training for facilitators. Several successful parenting group interventions have used facilitators with knowledge of psychological development (Enav et al., 2019; Havighurst et al., 2009; Suchman et al., 2008). There is less evidence in relation to the skills of showing fallibility and managing group dynamics, which may be due to difficulties in measuring these factors. However, the findings of the current study suggest that these skills are likely to contribute to a successful therapeutic alliance between parents and facilitators, leading to more successful outcomes.

The importance of parental peer support was identified as a theme across all three groups. However, in the survey, these themes scored more moderately in terms of consensus with the exception of ‘Relationships and support from other parents’. Similarly, modest consensus was achieved for themes related to creating a supportive network between parents outside of sessions. Previous research would suggest that relationships with parental peers and facilitators are an important element of a group parenting intervention (Garcia et al., 2018). It appears that relationships with facilitators and between parents are important, but could potentially mean that the evolution of these relationships should remain flexible. Therefore, the analysis would indicate that the intervention manual should highlight the potential benefits of peer support, but not make any mandatory recommendations, allowing facilitators to approach this flexibly, depending on the service context.

### Intervention content

There were many themes identified in terms of intervention content that also achieved high consensus at Round 2. Parent's emotional literacy, increasing caregiver knowledge of emotions and acceptance and normalising of emotion were rated highly in the consensus survey by all participants. Other components achieving high consensus (and therefore supporting their inclusion in the manual and intervention materials) were deepening understanding of behaviour, naming emotions and modelling, encouraging communication, linking emotions to bodily sensations and embedding emotional talk in everyday life. These factors appear to fit with existing literature around teaching children directly to learn about emotions (Hubble et al., 2015; Hunnikin et al., 2020; van Goozen, 2015; Webster‐Stratton & Reid, 2003).

Parents' understanding of their own emotions, and being able to regulate themselves, was also a theme which achieved high consensus. As such, the analysis indicated that skills and techniques related to helping parents understand their own emotions should be included in the intervention. This is in line with previous research (Hajal & Paley, 2020). The importance of safe and warm parental relationships to facilitate emotional learning, PRF and developing empathy for the child achieved high consensus for inclusion in the intervention. These findings fit with the existing mentalisation literature around optimal emotional development occurring in the context of a secure child–parent relationship and the evidence on PRF (Asen & Fonagy, 2012; Meins et al., 2013; Sharp et al., 2007).

Preparing parents for difficult content, actively managing the complexities of confidentiality, and supporting parents to understand that they can facilitate change in their children and indeed themselves, all received high consensus for inclusion. A theme that was identified across all groups but achieved only moderate consensus was around thinking about parents' own experiences of being parented. Therefore, on balance, the analysis suggested that enabling parents to reflect on their own experiences of being parented was an important element to include. However, that it should be done so sensitively, and in a non‐directive way, allowing parents to share as little or much as they wished to. Prior research would suggest that this is an important element for a parenting intervention related to understanding emotions (Asen & Fonagy, 2012; Camoirano, 2017).

### Limitations

The current study has several limitations. Firstly, the Delphi method can be applied in diverse and varied forms. In the first round of this study participants were sent a brief rationale and outline of the proposed intervention (as has been done in previous research; Lewis et al., 2013). The information provided to participants prior to the interview may have led to some bias in responses, and participants may have displayed performance effects, feeling unable to be honest about their views. However, it did appear that participants did give critiques and comments that were not solely acquiescing to the information provided.

In addition, the author was aware of their own biases in terms of beliefs about emotions and emotional expression as informed by living in a white western culture and prior reading and training on relevant theories and models. The research team identified these risks before commencing interviews, and the interviewer made concerted efforts to encourage critique and openness. An additional limitation was that all participants were of white ethnicity and majority female (only males were in the parent group). Therefore, this limits the range and diversity of views that were gathered.

Secondly, in this study, three distinct groups of participants were identified, which is not commonplace in a Delphi study. It is possible to question the expertise of parents and family intervention facilitators. However, there is increasing acknowledgement of the contribution and value of ‘experts by experience’ in Delphi studies, as well as evidence of high consensus agreement between experts by experience and professional groups (Jorm, 2015). MRC guidance (Craig et al., 2013) for developing and evaluating complex interventions highlights that stakeholders should be involved at all stages of the process. Therefore, the expertise of parents and facilitators was essential for informing the development of the intervention, and this appeared to be an innovative way of combining views.

Finally, Delphi studies often involve multiple rounds of questioning to build consensus. In the current study, only two rounds were used. However, given the high levels of consensus achieved in the second round, it appears this was adequate and is also in line with studies using a similar methodology (Domoney et al., 2020).

### Research implications

This research has informed the development of materials for a parenting intervention for parents with children experiencing emotional or behavioural difficulties. As the intervention has been developed with input from a wide range of groups and in line with best practice guidance, it is worthy of further research. As a further step in the development of this intervention, it would also be important to gather the views and recommendations of young people who the intervention aims to benefit. Due to the intervention being targeted at parents of children in early to middle childhood, it would be key that this was done in a developmentally appropriate manner using up‐to‐date guidance for including young people in research.

In line with the MRC Developing and Evaluating Complex Interventions Framework (Craig et al., 2013), initially, the intervention should undergo a feasibility and pilot trial, to inform whether and how the intervention should progress to a randomised control trial. Feasibility questions should include how to identify services and participants for recruitment, testing the acceptability of the intervention and surrounding procedures, assessing the suitability and acceptability of outcome measurements, and calculating appropriate sample sizes. It will also be important to examine parental factors that could predict engagement and treatment outcomes as well as considering adaptations that need to be made for parents with additional needs. Refinements to the intervention and trial design can therefore be made before progressing to larger scale studies.

### Clinical implications

The current study has informed the development of a promising brief parenting intervention to help parents to support their children to learn about emotions. There is prior evidence to suggest that enhancing emotional understanding could have a wide range of impacts on emotional and behavioural problems in children (Morris et al., 2010; Olson et al., 2011). This intervention could therefore be an efficacious and cost‐effective option for both families and services and answers calls for preventative programmes to address early emerging behavioural and emotional difficulties (Collishaw & Sellers, 2020; National Assembly for Wales, Children, Young People and Education Committee, 2018).

## CONCLUSIONS

This study aimed to systematically develop a brief parenting group‐delivered intervention aiming to improve children's understanding of emotions. Following MRC guidance and using a Delphi Survey method technique to gather consensus, the important elements of the intervention have been established. This includes a number of practical elements, ways in which a safe group space can be established and recommendations for intervention content. All items generated in the first round achieved at least moderate consensus (in the second round) for inclusion in the intervention. The study gathered consensus from a wide range of participants and has been used to inform the development of the intervention. The intervention is likely to be a beneficial intervention for parents supporting children with emotional and behavioural problems and will now go on to be researched further in the form of feasibility and pilot trial studies.

## AUTHOR CONTRIBUTIONS

**Sarah Lavender:** Writing – original draft; writing – review and editing; conceptualization; investigation; methodology; validation; formal analysis. **Chris Hobson:** Conceptualization; investigation; methodology; supervision; resources. **Cerith Waters:** Conceptualization; investigation; writing – review and editing; methodology; formal analysis; supervision; resources.

## CONFLICT OF INTEREST STATEMENT

The authors declare that there are no conflicts of interest with respect to the authorship or the publication of this article.

## ETHICS STATEMENT

Cardiff University Ethics Committee granted ethical approval for the research.

## Supporting information


Data S1.

## Data Availability

The data that support the findings of this study are available from the corresponding author upon reasonable request.

## APPENDIX A INTERVIEW SCHEDULES

### CLINICIANS AND ACADEMICS

1. In relation to the theoretical rationale, do you think in its current form it is understandable? Do you have any general comments?
  - Do you think there is a need for a short‐term parenting intervention specifically about encouraging their children to learn about emotions? (why/why not?)
  - What theories do you consider should drive such an intervention?
  - Would you suggest any changes to the overall rationale for the group?
  - If such a short‐term group could be developed that is effective in giving parents tools to encourage their children learning about emotions:
    - Which settings do you think it should/could be delivered, and by whom?
    - Would you foresee other methods of delivery to increase its impact (e.g., web/app‐based; self‐help guide/manual)
    - Do you feel that it should be delivered to targeted populations (e.g., parents of children with emotional or behavioural difficulties, or developed so that it is suitable to be offered universally?)
2. Do you think the broad aims of the group are helpful? Do you think any other aims should be included and any of the current aims should be removed?
3. What do you think about the basic set‐up of the intervention that has been proposed – timings, spacing etc.?
4. What do you think about the proposed content of Session 1? Do you have any additional ideas that you think should be included?
5. What do you think about the proposed content of Session 2? Do you have any additional ideas that you think should be included?
6. What do you think about the proposed content of Session 3? Do you have any additional ideas that you think should be included?
7. What do you think about the proposed content of Session 4? Do you have any additional ideas that you think should be included?
8. Do you have any additional games, activities or techniques that you think parents should be given during the group that would be effective and engaging in enabling their children to learn about emotions or to try out at home?

### PARENTS

1a. How many children do you have and what are their ages?

1b. Have you attended parenting groups before?

1c. What have you valued about the groups you have attended (if anything) and what have you found difficult (if anything)?

2. Following on from the outline you have read and my explanation of the group we are planning to develop, from your point of view, how interested would you be in attending a short‐term parenting intervention specifically about encouraging their children to learn about emotions? (why/why not?).

3. In general, what factors would make you likely to attend a parenting group?

4. What factors would make you unlikely to attend a parenting group?

5. What do you see as the advantages and disadvantages of an online parenting group or a face‐to‐face parenting group?

6. What setting, size of group and timings would you ideally like for a parenting group?

7. How do you feel about at‐home tasks between sessions?

8. Are you likely to feel comfortable to think about your own emotions and how you have been parented in a group?

9. Do you have any comments about anything else outlined in the document about the intervention?

### FACILITATORS

1. In your experience what are some of the key ingredients of an effective parenting intervention?
2. In your experience what are some of the best ways to encourage children to learn about emotions? Any activities, games, ideas etc.
3. Do you think there is a need for a short‐term parenting intervention specifically about encouraging their children to learn about emotions? (why/why not?)
4. If such a short‐term group could be developed that is effective in giving parents tools to encourage their children learning about emotions:
  - What settings do you think it should/could be delivered, and by whom?
  - What do you think about delivering the intervention online compared to face to face?
  - Do you feel that it should be delivered to targeted populations (e.g., parents of children with emotional or behavioural difficulties, or developed so that it is suitable to be offered universally?)
5. In your experience what are some of the common difficulties in delivering effective parenting interventions?
6. In your experience how willing are parents to reflect on their own experiences of being parented and their ability to understand and regulate their own emotions?
7. Do you have any comments about anything else outlined in the document about the intervention?
