# Supplementary material for: The development of a brief parenting intervention to improve children's understanding of emotions: A Delphi study
Source: Psychol Psychother. 2026 Feb 12;99(2):562–86. doi: 10.1111/papt.70043 (PMC13162189; doi:10.1111/papt.70043)
Supplement: Supplementary file 1 — Data S1. [file PAPT-99-562-s001.docx]

**Supplementary Information**

|  | **Authors** | **Participants and Design** | **Number of sessions** | **Delivery Location and Facilitators** | **Intervention** | **Outcome measures** | **Findings** |
| --- | --- | --- | --- | --- | --- | --- | --- |
| 1 | Peters et al., (2005) | N = 75 mothers with children aged 3-10 years with ODD and/ or CD  Observational | 10 two-hour weekly sessions | Information not provided | Parent Management Training that focuses on parenting behaviours social learning principles to help parents understand contingencies that maintain problem and prosocial behaviours. Specific techniques are taught to reinforce desirable behaviours and extinguish undesirable behaviours. | **Parent Measures**  Parental interviews using the Camberwell Family Interview methodology. Interview data was  independently coded using the Leeds Attributional Coding System.  **Child Measures**  None | Mothers who made child-responsibility attributions and were highly critical about their child’s behaviour were no more likely than non-blaming parents to drop out prematurely from the intervention. Expressing an understanding of their own role in managing their child’s behaviour was predictive of attendance. |
| 2 | Havighurst  et al., (2009) | N = 218 parents of  children aged 4.0–5.11 years  RCT, intervention vs. waitlist control group | 6 two-hour weekly sessions | Community settings  Facilitators were mental health professionals | Tuning Into Kids teaches parents emotion coaching skills to support children’s emotional and behavioural development. The aim is to help parents understand their own emotions, in order to better respond to their child’s emotions and to develop strategies for managing difficult emotions. | **Parent measures**  DERS  MESQ  PESQ  GHQ 28  **Child measures**  ECBI completed by parents and teachers | Results showed parents in the intervention condition reported significant increases in emotion coaching and significant reductions in the dismissing of emotions with their children. Child behaviour was also reported to improve. Of those with clinical levels of behaviour difficulties, more than half were no longer at clinical level post intervention. |
| 3 | Havighurst et al., (2010) | N = 216 parents of children aged 4.0–5.11 years.  RCT, intervention vs. waitlist control group | 6 two-hour weekly sessions.  Two bimonthly  booster sessions after the initial six weeks. | Community settings  Facilitators were mental health professionals | Tuning Into Kids teaches parents emotion coaching skills to support child’s emotional development and behaviour. Helps parents understand their own emotions, respond to their child’s emotions and strategies for managing difficult emotions. | **Parent measures**  DERS  MESQ  *A structured parent–child story‐telling task* ([Cervantes & Callanan, 1998](https://onlinelibrary.wiley.com/doi/full/10.1111/j.1469-7610.2010.02303.x#b2)) was completed to directly assess parent emotion coaching.  **Child measures**  PPVT-III  EST  ECBI completed by parents and teachers. | Parents in the intervention condition reported significant improvements in their own emotional awareness and regulation, increases in emotion coaching, and decreases in emotionally dismissive beliefs and behaviours. There were increases in parents observed use of emotion labels and discussion of causes and consequences of emotions with their children. Child emotional knowledge improved, and reductions in child behaviour problems were reported by parents and teachers. |
| 4 | Gaviţa et al.,  (2012) | N = 97 Romanian foster parents and children aged 5-18 years.  RCT, intervention vs. waitlist control group | 4 four-hour weekly sessions and a three-month  follow-up session | Not reported | A brief enhanced cognitive–  behavioural parenting program that teaches parenting skills, functional emotion-regulation strategies, and unconditional acceptance of the self (parent) and child. | **Parent Measures**  PS  PED  **Child Measures**  Placement disruption  CBCL | Results supported the efficacy of the program, in treating child behaviour problems and reducing parental emotional distress. No between group differences in  placement stability. |
| 5 | Wilson et al., (2012) | N = 128 parents of children aged 4.0 –5.11 years  RCT, intervention vs. waitlist control group | 6 two-hour weekly sessions | Community settings  Facilitators were mental health professionals | Tuning Into Kids teaches parents emotion coaching skills to support child’s emotional development and behaviour. Helps parents understand their own emotions, respond to their child’s emotions and strategies for managing difficult emotions. | **Parent measures**  MESQ  CCNES  APQ  APC  **Child measures**  ECBI  DECA  SCBE-30 | Intervention parents were  significantly less emotionally dismissive and more coaching in their practices in response to children’s negative emotions. Improvements in both conditions for parent-reported child behaviour and teacher-reported social  competence and reduction  in number of behaviour problems. |
| 6 | Havighurst et al., (2013) | N = 54 parents  of 4–5-year-old children who presented with externalizing behaviour difﬁculties  RCT, intervention vs. waitlist control group | 6 two-hour weekly sessions | Community settings  Facilitators were mental health professionals | Tuning into Kids teaches parents emotion coaching skills to support child’s emotional development and behaviour. Helps parents understand their own emotions, respond to their child’s emotions and strategies for managing difficult emotions. | **Parent Measures**  MESQ  A structured parent–child story‐telling task ([Cervantes & Callanan, 1998](https://onlinelibrary.wiley.com/doi/full/10.1111/j.1469-7610.2010.02303.x#b2)) was completed to directly assess parent emotion coaching.  **Child Measures**  PPVT‐III  EST  ECBI | Parents in both conditions reported reduced emotional dismissiveness and child behaviour problems. In the intervention group, parents also reported greater empathy and improved observed emotion coaching skills; their children had greater emotion knowledge and reduced teacher-reported behaviour problems. |
| 7 | Herbert et al.,  (2013) | N = 31 Hyperactive pre-schoolers – 31 children – 31 mothers and 18 parents  RCT, intervention vs. waitlist control group | 14 one and a half hour weekly sessions | University based community mental health clinic  Facilitators were Clinical Psychology Doctoral students or licensed Psychologists | Parenting your hyperactive pre-schooler teaches traditional parenting and emotion socialisation strategies | Audiotaped Assessment of Parent-Child Interaction  **Parent measures**  PS  CCNES  **Child measures**  BASC 2-PRS  DBRS  ERC | Mothers reported less child inattention, hyperactivity, defiance and emotional lability. Results also showed less negative parenting practices. |
| 8 | Valentino et al., (2013) | N = 44 parent-child dyads of maltreating parents and 3–6 years old children  RCT, intervention vs. waitlist control group | 4 one-hour weekly in-home training sessions | In home  Facilitators were bachelor level family coaches | Reminiscing and Emotion Training (RET) is a relational intervention involving training parents to have more sensitive and elaborate conversations about past emotional events and activities to help parents and children with emotion identification. | Parent–child memory conversations (Fivush, et al., 2006; Salmon et al, 2009) **Child measures**  Experimenter-child memory conversations (Reese and Newcombe, 2007)  PPVT-III | Parents in the RET condition provided more high-elaborative utterances, references to children’s negative emotions, and explanations of children’s emotion during reminiscing than parents in the control condition. Children in the reminiscing condition had richer memory recall and made more emotion references than did children in the control condition during reminiscing with their mothers, but not with an experimenter. |
| 9 | David, David & Dobrean., (2014) | N = 130 parents with children aged 4 – 12 years.  RCT, standard CBT group vs. Enhanced CBT group | 10 ninety-minute weekly sessions. | Schools or kindergartens  No details of facilitators provided | Rational Positive Parenting (enhanced CBT) or standard cognitive behavioural program. The enhanced program incorporated two sessions specifically focused on teaching parents’ strategies for emotion regulation in addition to standard cognitive and behavioural strategies. | **Child measures**  CBCL  C-TRF  TRF  The Child Behaviour Checklist and Caregiver-Teacher Report Form/ Teacher Report Form | Decreases in child externalizing symptoms in standard and enhanced programs with greater improvements in the enhanced program at 1 month follow-up. |
| 10 | Kehoe et al., (2014) | N = 225 parents of children aged 10-13 years.  RCT, intervention vs. waitlist control group | 6 two-hour weekly sessions | Local community centres  Facilitators were mental health professionals or psychology graduates who had undergone training in the intervention. | Tuning Into Teens teaches parents skills to better understand and support teenagers’ emotion aiming to foster parent-adolescent emotional competence and communication. | **Parent Measures**  GHQ 28  DERS  EAC parent version  SCAS-P  **Child Measures**  EAC youth version   1. SCAS | Multilevel analyses showed  significant improvements in parental emotion socialization and reductions in youth  internalizing difficulties for the intervention condition. |
| 11 | Lauw et al.,  (2014) | N = 34 parents of toddlers aged 18 to 36  Months  Pilot study | 6 two-hour weekly sessions | Community settings  Facilitators were mental health professionals | Tuning in to Toddlers (TOTS) teaches emotion coaching skills to help parents improve their own emotion regulation and help toddlers develop emotional competence | Parent-Child Observation Tasks  **Parent measures**  MESQ  CTNES  **Child measures**  Brief Infant-Toddler Social and Emotional Assessment (Briggs-Gowan & Carter, 2007) | Results showed significant increases in self-reported and observed parental emotion  coaching behaviours and the use of emotion talk after intervention. There was a significant decrease in self-reported and observed emotion dismissing  behaviours, and toddler externalizing behaviour problems. |
| 12 | Rodríguez et al., (2014) | N = 28 mothers with children of mean age of 37.79 months  Pilot RCT, intervention vs. waitlist control group | 1 hour a week – number of sessions not stated | Neonatal follow-up clinic  No details of facilitators provided | Parent-Child Interaction Therapy (PCIT) helps parents to learn how to follow their child’s lead in play and use differential attention to increase positive behaviours and decrease disruptive behaviours. Coached by therapists through a one-way mirror. | **Child measures**  ECBI  Emotion regulation was measured using a behavioural coding scheme | Improved emotion regulation for participants in the intervention condition. Higher levels of emotion dysregulation at baseline were associated with greater improvements in child disruptive behaviour following the intervention. |
| 13 | Bammens et al., (2015) | N = 22 foster parents  NRCT, intervention vs treatment as usual (4 hours of lecture information) | 3 three-hour sessions over 4-6 weeks | Community setting  Facilitator had a background working in foster care as well as training in mentalization- based interventions. | Family Minds is a psycho-educational and interactive programme which includes elements of mentalisation-based family therapy, lectures, group exercises and homework. | **Parent measures**  FMSS coded for reflective functioning | Significant increase in reflective functioning on the FMSS in the intervention group compared to the control group. |
| 14 | Havighurst  et al., (2015) | N= 204 primary caregivers and their children aged of 5-9 years  Observational (No control) | 8 two-hour weekly sessions | Community settings  Facilitators were mental health professionals | Tuning in to Kids teaches parents emotion coaching skills to support child’s emotional development and behaviour. Helps parents understand their own emotions, respond to their child’s emotions and strategies for managing difficult emotions. | **Parent Measures**  MESQ  **Child Measures**  ECBI  KAI-R  SDQ  SCRS | Results showed intervention parents but not controls became less emotionally dismissive and increased in empathy, and children showed better emotion understanding and behaviour compared to control children. |
| 15 | Ravindran et al., (2015) | N = 84 parents with at least two children between 4 and 8 years old  RCT, intervention vs. waitlist control group | 4 one-hour weekly sessions | University research facility  No details of facilitators provided | More Fun with Sisters and Brothers Program (MFWSB) teaches parents how to help their children learn and practice core emotion regulation skills. | **Parent measures**  Parental Emotion Regulation in the Sibling Context Questionnaire (Kramer et al., 2025)  ERQ  PEPC-SRQ | Mothers in the intervention group reported lower levels of dysregulation and suppression and higher levels of reappraisal. Also, lower levels of maternal and paternal negative reactivity in the sibling context in the intervention group. |
| 16 | Chronis-Tuscano et al., (2016) | N = 9 parents with children 3-7 years old with Attention Deficit Hyperactivity Disorder  Case series | Three 60–90-minute child-directed  interaction (CDI) and three 60–90-minute parent-directed interaction  (PDI) sessions. | ADHD Clinic  Facilitators were masters’ clinicians with additional training on PCIT | Parent–child interaction  therapy – Emotional Development (PCIT-ED) - adaptation of PCIT which teaches parents emotion coaching skills including PRIDE skills (praise, reflection, imitation,  description, enthusiasm). | **Parent Measures**  The Dyadic Parent–Child Interaction Coding System  (DPICS; Eyberg, Nelson, Duke, & Boggs, 2004)  **Child measures**  DBD  IRS  CBCL – parent and teacher  ERC | Intervention led to improvements in children’s externalising behaviour and improved positive parenting behaviours. No change seen in parent-child interactions. |
| 17 | Duncombe et al., (2016) | N = 320 parents with children aged 4-9 years old with disruptive behaviour problems  RCT with 3 conditions. Tuning into kids, a multisystemic intervention and a waitlist control group | 8 two-hour weekly sessions week | Community settings  Facilitators were mental health professionals | Tuning into Kids teaches parents emotion coaching skills to support child’s emotional development and behaviour. Helps parents understand their own emotions, respond to their child’s emotions and strategies for managing difficult emotions. | **Child measures**  ECBI  SDQ  A structured home interview with child | The results indicated that the multisystemic and the tuning into kids interventions were equally effective compared to the waitlist control group in reducing child conduct problems. |
| 18 | Mason et al,.  (2016) | N = 321 parents of children with mean age of 13.41 years old  RCT, intervention vs. minimal contact control group | 6 two-hour weekly sessions  8 two-hour weekly sessions | Community setting  No details of facilitators provided | Common Sense Parenting Program (CSP) teaches parents skills to improve children’s behaviour through boundary setting, positive reinforcement and respectful communication.  CSP plus has two additional sessions focused on helping parents support children to move towards independence and school transitions. | **Child measures**  SDQ  SCRS  Questions on substance use | Neither intervention had statistically significant total effects on the child outcome measures. CSP had statistically significant indirect effects on reduced substance use and school suspensions at the 1- and 2-year follow-up through increased child emotion regulation skills. |
| 19 | Martin et al., (2017) | N = 4 mothers with severe emotion dysregulation with a diagnosis of a mental health disorder  Case series | 22 two and a half hours weekly sessions | Mental Health setting  No details of facilitators provided | Dialectical Behaviour Therapy group based on the DBT Skills Training Manual (Linehan, 2015b) that includes modules on mindfulness, distress tolerance, emotion regulation and interpersonal effectiveness. | **Parent measures**  DBT diary card  Parental interviews  PSS  CCNES  DERS  BDI-II | DBT skills training reduced mental health and emotion dysregulation symptoms. DBT skills were used in parenting context nearly half of the time and were related to improvements in parenting behaviours and parental stress. |
| 20 | Adkins, Luyten & Fonagy. (2018) | N = 102 foster parents  NRCT, intervention vs treatment as usual (usual foster parent class) | 3 three-hour sessions over 4-6 weeks | Community setting  Facilitator had a background working in foster care as well as training in mentalization-based interventions. | Family Minds is a psycho-educational and interactive programme which includes elements of mentalisation-based family therapy, lectures, group exercises and homework. | **Parent measures**  PRFQ  FMSS coded for reflective function  PSI-SF | The intervention increased reflective functioning and reduced caregiver stress in foster parents. |

**Key:** DERS (Difficulties in Emotion Regulation Scale, Gratz & Roemer, 2004); MESQ (Maternal Emotional Style Questionnaire, Lagacé‐Séguin & Coplan, 2005); PPVT-III (Peabody Picture Vocabulary Test, Dunn & Dunn, 1997); ECBI (Eyberg Child Behavior Inventory, Eyberg & Pincus, 1999)

GHQ-28 (General Health Questionnaire, Goldberg, 1981); EAC (Emotions as a Child scale, Magai, 1996); SCAS-P (Spence Child Anxiety Scale for parents, Nauta et al, 2004); SCAS (Spence Children’s anxiety scale, Spence, 1998); EST (Emotion Skills Task, Denham, 1986); KAI-R (Kusche Affective Inventory, Kusche, Greenberg & Beilke, 1988); SDQ (Strengths and Difficulties Questionnaire, Goodman, 1997); SCRS (Social Competence Rating Scale, Gifford-Smith, 2000); PS (Parenting Scale, Arnold et al., 1993); PED (Profile of Emotional Distress, Opris & Macavei, 2007); CBCL (Child Behaviour Checklist, Achenback, 1991); PESQ (Parent Emotional Style Questionnaire, Havighurst et al., 2010); ERQ (Emotion Regulation Questionnaire, Gross & John, 2003); PEPC-SRQ (Parental Expectations and Perceptions of Children’s Sibling Relationships Questionnaire, Kramer & Baron, 1995); CTNES (Coping with Toddlers’ Negative Emotions Scale, Spinrad et al., 2004); CCNES (Coping With Children’s Negative Emotion Scale, Fabes, Eisenberg & Bernzweig, 1990); DBRS (Disruptive Behavior Rating Scale, Barkley & Murphy, 1998); ERC (Emotion Regulation Checklist, Shields & Cicchetti, 1997); BASC-2 (Behavior Assessment System for Children Second Edition, Reynolds & Kamphaus, 2004); PSS (Parenting Stress Scale, Berry & Jones, 1995); BDI-II (Beck Depression Inventory, Beck, Steer & Brown, 1996); DBD (Disruptive Behavior Disorders, Pelham, Gnagy, Greenslade & Milich, 1992); IRS (Impairment Rating Scale, Fabiano et al., 2006); C-TRF (Caregiver-Teacher Report Form, Achenbach, 1991); TRF (Teacher Report Form, Achenbach, 1991); APQ (Alabama Parenting Questionnaire, Shelton, Frick & Wootton, 1996); DECA (Devereux Early Childhood Assessment, LeBuffe & Naglieri, 1999); SCBE-30 (Social Competence and Behavior Evaluation, LaFreniere & Dumas, 1995); PRFQ (Parental Reflective Functioning Questionnaire, Luyten et al., 2017); SPSQ (Swedish Parenthood Stress Questionnaire, Östberg et al, 1995); EPDS (Edinburgh Postnatal Depression Scale, Cox, Holden & Sagovsky, 1987); CES-D (Center for Epidemiological Studies-Depression Scale, Radloff, 1977); PBI (Parental Bonding Index, Parker et al., 1979); BITSEA (Brief Infant Toddler Social Emotional Assessment, Briggs-Gowan & Carter, 2006); EA-SR (Emotional Availability – Self Report, Vliegen et al, 2009); FMSS (Five-minute speech sample, Magana et al., 1986); PSI-SF (Parenting Stress Index – Short Form, Abidin, 1995); RCT (Randomised Control Trial); NRCT (Non-Randomised Control Trial); ODD (Oppositional Defiance Disorder; CD (Conduct Disorder).

**References**

1. Peters, S., Calam, R., & Harrington, R. (2005). Maternal attributions and expressed emotion as predictors of attendance at parent management training. *Journal of Child Psychology and Psychiatry*, *46*(4), 436-448.
2. Havighurst, S. S., Wilson, K. R., Harley, A. E., & Prior, M. R. (2009). Tuning in to kids: an emotion‐focused parenting program—initial findings from a community trial. *Journal of Community Psychology*, *37*(8), 1008-1023.
3. Havighurst, S. S., Wilson, K. R., Harley, A. E., Prior, M. R., & Kehoe, C. (2010). Tuning in to Kids: improving emotion socialization practices in parents of preschool children–findings from a community trial. *Journal of Child Psychology and Psychiatry*, *51*(12), 1342-1350
4. Gaviţa, O. A., David, D., Bujoreanu, S., Tiba, A., & Ionuţiu, D. R. (2012). The efficacy of a short cognitive–behavioral parent program in the treatment of externalizing behavior disorders in Romanian foster care children: Building parental emotion-regulation through unconditional self-and child-acceptance strategies. *Children and Youth Services Review*, *34*(7), 1290-1297.
5. Wilson, K. R., Havighurst, S. S., & Harley, A. E. (2012). Tuning in to Kids: An effectiveness trial of a parenting program targeting emotion socialization of preschoolers. *Journal of Family Psychology*, *26*(1), 56.
6. Havighurst, S. S., Wilson, K. R., Harley, A. E., Kehoe, C., Efron, D., & Prior, M. R. (2013). “Tuning into Kids”: Reducing young children’s behavior problems using an emotion coaching parenting program. *Child Psychiatry & Human Development*, *44*(2), 247-264.
7. Herbert, S. D., Harvey, E. A., Roberts, J. L., Wichowski, K., & Lugo-Candelas, C. I. (2013). A randomized controlled trial of a parent training and emotion socialization program for families of hyperactive preschool-aged children. *Behaviour Therapy*, *44*(2), 302-316.
8. Valentino, K., Comas, M., Nuttall, A. K., & Thomas, T. (2013). Training maltreating parents in elaborative and emotion-rich reminiscing with their preschool-aged children. *Child Abuse & Neglect*, *37*(8), 585-595.
9. David, O. A., David, D., & Dobrean, A. (2014). Efficacy of the rational positive parenting program for child externalizing behavior: can an emotion-regulation enhanced cognitive-behavioral parent program be more effective than a standard one?. *Journal of Evidence-Based Psychotherapies*, *14*(2), 159.
10. Kehoe, C. E., Havighurst, S. S., & Harley, A. E. (2014). Tuning in to teens: Improving parent emotion socialization to reduce youth internalizing difficulties. *Social Development*, *23*(2), 413-431.
11. Lauw, M. S., Havighurst, S. S., Wilson, K. R., Harley, A. E., & Northam, E. A. (2014). Improving parenting of toddlers’ emotions using an emotion coaching parenting program: A pilot study of tuning in to toddlers. *Journal of Community Psychology*, *42*(2), 169-175.
12. Rodríguez, G. M., Bagner, D. M., & Graziano, P. A. (2014). Parent training for children born premature: A pilot study examining the moderating role of emotion regulation. *Child Psychiatry & Human Development*, *45*(2), 143-152.
13. Bammens, A. S., Adkins, T., & Badger, J. (2015). Psycho-educational intervention increases reflective functioning in foster and adoptive parents. *Adoption & Fostering*, *39*(1), 38-50.
14. Havighurst, S. S., Duncombe, M., Frankling, E., Holland, K., Kehoe, C., & Stargatt, R. (2015). An emotion-focused early intervention for children with emerging conduct problems. *Journal of abnormal child psychology*, *43*(4), 749-760.
15. Ravindran, N., Engle, J. M., McElwain, N. L., & Kramer, L. (2015). Fostering parents’ emotion regulation through a sibling-focused experimental intervention. *Journal of Family Psychology*, *29*(3), 458.
16. 16. Chronis-Tuscano, A., Lewis-Morrarty, E., Woods, K. E., O’Brien, K. A., Mazursky-Horowitz, H., & Thomas, S. R. (2016). Parent–child interaction therapy with emotion coaching for preschoolers with attention-deficit/hyperactivity disorder. *Cognitive and Behavioral Practice*, *23*(1), 62-78.
17. Duncombe, M. E., Havighurst, S. S., Kehoe, C. E., Holland, K. A., Frankling, E. J., & Stargatt, R. (2016). Comparing an emotion-and a behavior-focused parenting program as part of a multsystemic intervention for child conduct problems. *Journal of Clinical Child & Adolescent Psychology*, *45*(3), 320-334.
18. Mason, W. A., January, S. A. A., Fleming, C. B., Thompson, R. W., Parra, G. R., Haggerty, K. P., & Snyder, J. J. (2016). Parent training to reduce problem behaviors over the transition to high school: Tests of indirect effects through improved emotion regulation skills. *Children and youth services review*, *61*, 176-183.
19. Martin, C. G., Roos, L. E., Zalewski, M., & Cummins, N. (2017). A dialectical behavior therapy skills group case study on mothers with severe emotion dysregulation. *Cognitive and Behavioral Practice*, *24*(4), 405-415.
20. Adkins, T., Luyten, P., & Fonagy, P. (2018). Development and preliminary evaluation of family minds: a mentalization-based psychoeducation program for foster parents. *Journal of Child and Family Studies*, *27*(8), 2519-2532.
